# Peptide Aptamer–Paclitaxel Conjugates for Tumor Targeted Therapy

**DOI:** 10.3390/pharmaceutics17010040

**Published:** 2024-12-30

**Authors:** Xinyang Shen, Yuan Ma, Hang Luo, Razack Abdullah, Yufei Pan, Yihao Zhang, Chuanxin Zhong, Baoting Zhang, Ge Zhang

**Affiliations:** 1 Department of Obstetrics and Gynecology, Nanfang Hospital, Southern Medical University, Guangzhou 510515, China; 2 School of Chinese Medicine, Faculty of Medicine, The Chinese University of Hong Kong, Hong Kong SAR 999077, China; mayuan@hkbu.edu.hk (Y.M.);; 3 Law Sau Fai Institute for Advancing Translational Medicine in Bone &Joint Diseases, School of Chinese Medicine, Hong Kong Baptist University, Hong Kong SAR 999077, China

**Keywords:** cancer, paclitaxel, peptide aptamer, peptide aptamer–paclitaxel conjugate, therapeutic effect

## Abstract

**Background/Objectives**: Traditional paclitaxel therapy often results in significant side effects due to its non-specific targeting of cancer cells. Peptide aptamer–paclitaxel conjugates present a promising alternative by covalently attaching paclitaxel to a versatile peptide aptamer via a linker. Compared to antibody–paclitaxel conjugates, peptide aptamer–paclitaxel conjugates offer several advantages, including a smaller size, lower immunogenicity, improved tissue penetration, and easier engineering. **Methods**: This review provides an in-depth analysis of the multifunctional peptide aptamers in these conjugates, emphasizing their structural features, therapeutic efficacy, and challenges in clinical applications. **Results**: This analysis highlights the potential of peptide aptamer–paclitaxel conjugates as a novel and effective approach for targeted cancer therapy. By harnessing the unique properties of peptide aptamers, these conjugates demonstrate significant promise in improving drug delivery efficiency while reducing the adverse effects associated with traditional paclitaxel therapy. **Conclusions**: The incorporation of peptide aptamers into paclitaxel conjugates offers a promising pathway for developing more efficient and targeted cancer therapies. However, further research and clinical studies are essential to fully unlock the therapeutic potential of these innovative conjugates and enhance patient outcomes.

## 1. Introduction

**Figure 1:**
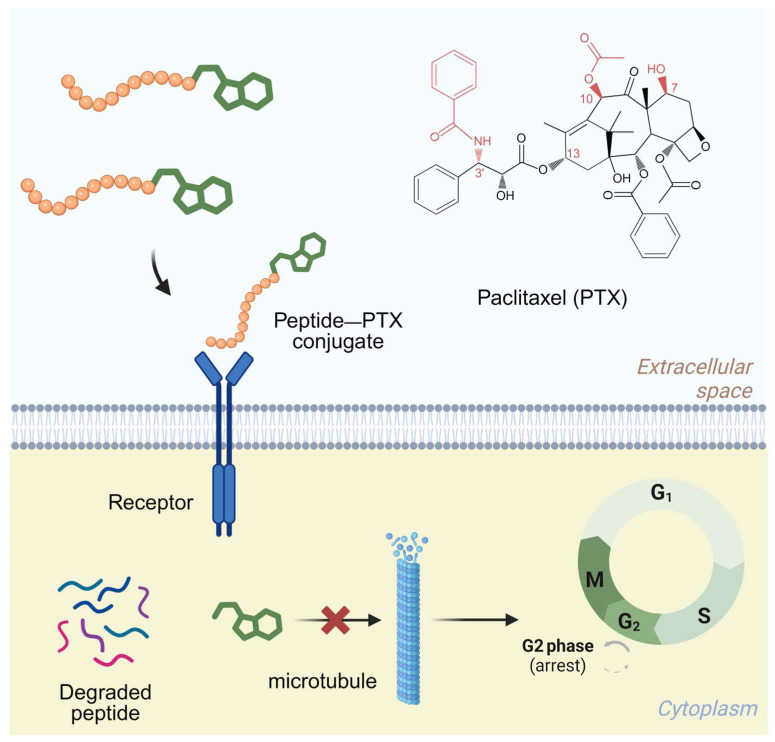
Schematic illustration depicting the mechanisms of PAPC in cancer cells. Created in BioRender.

Traditional chemotherapy frequently causes severe adverse effects due to its lack of specificity [1]. Targeted therapy has emerged as a promising strategy to improve treatment efficacy while reducing side effects [2,3]. Paclitaxel (PTX) prodrugs designed for specific tumors can be classified into small-molecule–PTX conjugates (SMDCs), antibody–PTX conjugates (ADCs), and aptamer–PTX conjugates (ApDCs). For instance, both the paclitaxel–lipoate conjugate (IDD-1040) and the docetaxel–biotin conjugate (IDD-1010) showed better effectiveness than free paclitaxel [4,5]. Peptide aptamers and natural products play vital roles in targeted cancer therapy [6,7]. The creation of peptide-aptamer–paclitaxel conjugates (PAPCs) epitomizes a state-of-the-art approach, merging the benefits of the simple synthesis of SMDCs with the precise targeting of ADCs [8]. Peptide aptamers, short synthetic peptides with exceptional affinity and specificity for their targets [9], facilitate precise molecular targeting of proteins that are overexpressed in cancer cells. This distinctive tool enables the direct delivery of paclitaxel to tumor tissues, enhancing therapy precision and efficacy (Figure 1) [10].

Proteins that are significantly upregulated in cancer can be categorized into various groups, including membrane receptors, cell adhesion molecules, extracellular matrix proteins, immune checkpoint proteins, and intracellular regulators. Membrane receptors, which play a vital role in controlling cellular signaling pathways, are frequently overexpressed in cancer [11]. Cell adhesion molecules and extracellular matrix proteins play pivotal roles in the tumor microenvironment, impacting tumor growth, invasion, and metastasis [12]. By targeting these molecules with PAPCs, the disruption of the tumor microenvironment can be achieved, thereby intensifying the cytotoxic effects on the cancer cells. Additionally, immune checkpoint proteins and intracellular regulators are crucial in modulating the immune response against cancer cells [13]. By targeting these molecules with PAPCs, not only can direct cytotoxicity be induced in cancer cells, but the immune microenvironment can also be modulated to bolster anti-tumor immune responses. This review encompasses research on a range of peptide aptamers used in paclitaxel targeted therapy, providing a comprehensive overview of their structures, therapeutic effects, and translational challenges.

## 2. Multi-Functional Linkers in PAPCs

**Figure 2:**
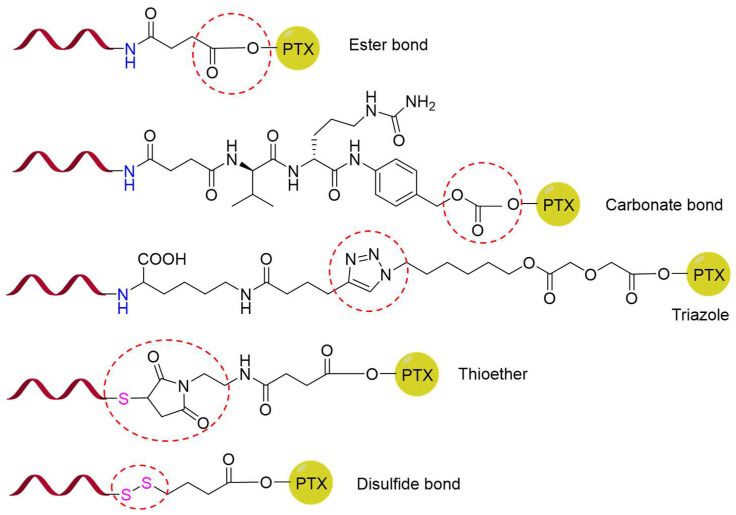
Schematic illustration depicting the linkers of PAPCs. The red circle represents different linkers used in PAPCs.

For paclitaxel to exert its activity, the C2′-OH linked to the C-13 side chain must remain unaltered. Consequently, modifications that mask the C2′-OH group can render paclitaxel inactive [8]. A specific peptide aptamer binds covalently to the C2′-OH of paclitaxel via a linker to form PAPC. In the synthesis process, the linker establishes two chemical connections: one between the peptide aptamer and the linker and the other between the linker and PTX. An ideal linker should demonstrate stability under typical physiological conditions while also being readily cleaved to release PTX within the tumor microenvironment. Moreover, the bond between the peptide aptamer and the linker should not affect the peptide’s affinity with its receptor [14]. Common functional groups found in the linker include acid-cleavable groups (ester, carbonate), enzyme-cleavable groups (ester, amido), redox-cleavable groups (disulfide bond), and non-cleavable groups (triazole, thioether) (Figure 2).

## 3. Multi-Functional Peptide Aptamers in PAPCs

Peptide aptamers, consisting of 5–20 amino acid residues, are engineered to specifically recognize receptors overexpressed on the surfaces of cancer cells [15,16]. This targeted interaction triggers receptor-mediated endocytosis, facilitating the internalization of PAPCs into the tumor cells. To enhance stability and optimize pharmacokinetics, peptide aptamers are often modified through hydrophobic alterations, covalent modifications, or long-chain fatty acid conjugation, which improve their binding affinity to human serum proteins [17,18,19,20]. The screening of peptide aptamers can be achieved using various methodologies, including conventional yeast two-hybrid assays [21], ribosome display [22], phage display [23], mRNA display [24], molecular docking simulations, and machine learning algorithms [25,26]. Conjugating paclitaxel to peptide aptamers enables precise drug delivery to tumor tissues, thereby enhancing the therapeutic efficacy and minimizing side effects [27]. The peptide aptamers employed in paclitaxel (PTX)-based anticancer strategies are summarized in Table 1.

### 3.1. Membrane Receptors Targeting Peptide Aptamers

#### 3.1.1. Low-Density Lipoprotein Receptor-Related Protein-1 (LRP-1)

**Figure 3:**
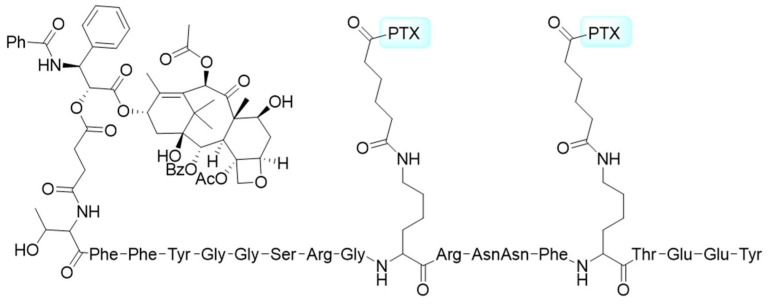
The structure of Angiopep-2 peptide–PTX conjugate.

Low-density lipoprotein receptor-related protein-1 (LRP1) is frequently present in human glioma cells and the blood-brain barrier (BBB), which makes it a promising target for treating glioblastoma [77,78]. Angiopep-2 (TFFYGGSRGKRNNFKTEEY), known for its specific binding to LRP-1, has been utilized as a ligand for delivering various chemotherapeutics. Régina et al. explored a novel drug delivery strategy to enhance the transport of paclitaxel (PTX) to the brain by conjugating it with Angiopep-2 through cleavable succinyl ester linkers (named ANG1005, Figure 3). Their research demonstrated that the conjugate exhibited enhanced brain penetration compared to free PTX. Moreover, ANG1005 displayed comparable or superior anticancer activity to PTX in inhibiting human cancer cells and human tumor xenografts. In preclinical studies, ANG1005 significantly increased survival rates in mice implanted with NCI-H460 lung carcinoma and U87 MG glioblastoma cells in the brain [28]. During phase I trials, ANG1005 showed favorable safety and tolerability profiles while also demonstrating efficacy in treating advanced solid tumors and brain metastases [79]. In a phase II study, ANG1005 exhibited anti-tumor activity both intracranially and extracranially. Notably, patients with leptomeningeal carcinomatosis experienced prolonged overall survival (OS) compared to historical controls, along with an improvement in clinical symptoms, even in this poor-prognosis population [80]. Currently, a phase III randomized, open-label trial is being conducted to assess the effectiveness of ANG1005 in patients with HER2-negative breast cancer [81].

Drappatz et al. reported additional therapeutic benefits of the conjugate, highlighting its good tolerability in clinical trials while achieving effective therapeutic concentrations for targeted tumor treatment [82]. Attaching polyethylene glycols (PEGs) to proteins or drug delivery nanosystems is a widely used technique to enhance the therapeutic efficacy of complex nano-biopharmaceuticals. However, these treatments often provoke immune responses, leading to the development of anti-drug antibodies (ADAs) [83]. To address this, Angiopep-2-modified PEG-co-poly(ε-caprolactone) nanoparticles (ANG-PEG-NPs) encapsulating PTX were developed. These nanoparticles demonstrated the ability to traverse the blood-brain barrier (BBB) and exhibited superior distribution, retention in 3D spheroids, and cellular uptake compared to unmodified nanoparticles (NPs) [84,85]. Furthermore, combining Angiopep-2 with cell-penetrating peptides, such as TAT, significantly enhanced their therapeutic efficacy. For instance, nanoparticles dual-functionalized with Angiopep-2 and TAT markedly improved PTX delivery to the brain [77]. Additionally, the receptor-associated protein (RAP) truncation peptide (RAP12, EAKIEKHNHYQK) demonstrated high specificity for LRP-1. As a result, RAP12-functionalized PTX-encapsulated PEG–PLA micelles (RAP12-PEG-PLA/PTX) effectively inhibited glioma growth and angiogenesis. Compared to the unmodified nanoparticles and free Taxol, RAP12-PEG-PLA/PTX significantly extended the median survival time in glioma-inoculated mouse models [29].

#### 3.1.2. Integrin

**Figure 4:**
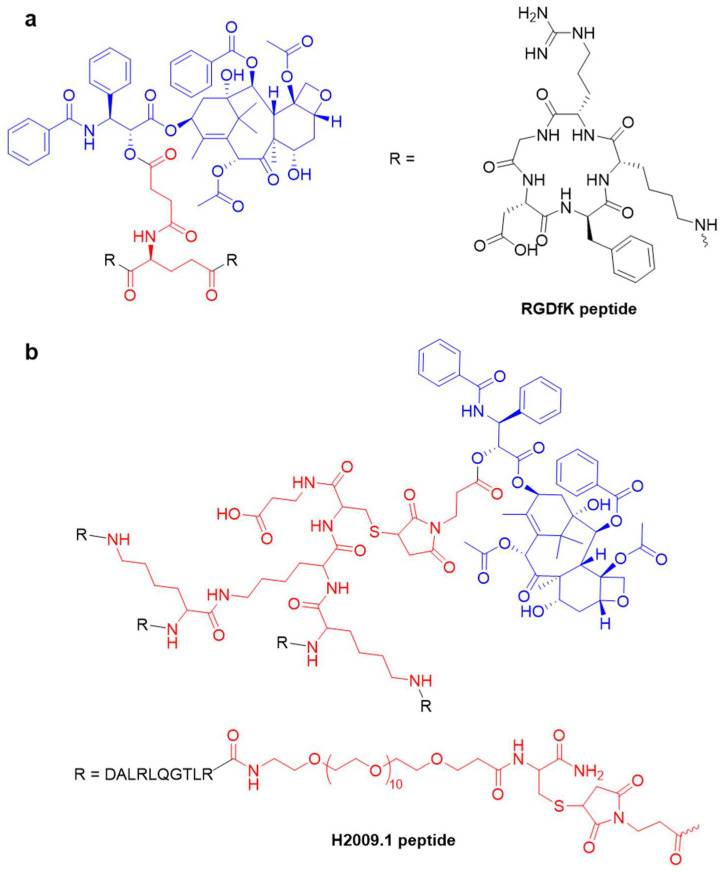
The structures of dimeric peptide–PTX conjugate (**a**) and tetrameric peptide–PTX conjugate (**b**). The integrin-targeting peptides were RGDfK and H2009.1, respectively.

Integrins play critical roles in cell adhesion, migration, and signaling. Among them, the α_v_β_3_ integrin is highly expressed in various cancers, including osteosarcomas, neuroblastomas, glioblastomas, malignant melanomas, and breast, lung, and prostate cancers [86,87]. Peptides containing the arginine–glycine–aspartic acid (RGD) motif exhibit a high affinity for α_v_β_3_ integrin, making them effective ligands for tumor-specific targeting (Figure 4a) [88]. RGD-conjugated PTXs have shown enhanced cellular uptake and therapeutic efficacy [37,38,89,90]. Notably, cyclic peptides such as _c_(RGDyK) show superior tumor specificity and binding affinity compared to linear RGD peptides. For example, Gd-liposomal nanoparticles (NPs) coated with _c_(RGDyK) achieved a 16-fold increase in MRI T1 relaxation in tumor cells [91]. The _c_(RGDKLAK)–PTX conjugate exhibited excellent solubility in water and tumor-growth inhibitory effects in glioblastoma-bearing mice [90]. Innovative strategies like _c_(RGDfK)-functionalized NPs incorporating pH-responsive cell-penetrating peptides (CPP), TRAIL, and PTX have demonstrated significant inhibition of tumor growth by up to 93.8% [92]. Furthermore, Raffaele et al. reported that _c_(DKP-RGD)–PTX conjugates exhibited superior efficacy compared to free PTX in a platinum-resistant ovarian cancer (IGROV-1/Pt1) xenograft mouse model, despite being administered at nearly half the molar dosage [93]. To further enhance cellular uptake, the RGD peptide was conjugated with the tissue-penetrating motif CendR to create iRGD (_c_[CRGDKGPDC]).iRGD–PTX NPs internalize via integrin αV-mediated endocytosis, resulting in significantly improved selective delivery efficacy and antitumor activity [35]. Combining iRGD with ultrasound irradiation or lipophilic PTX prodrugs has further enhanced the specificity and effectiveness of PTX delivery to tumor sites [94,95,96].

Emerging evidence underscores the importance of multi-targeted delivery systems for PTX to achieve optimal tumor specificity [97]. For instance, combining RGD with magnetic targeting increased the accumulation of PTX-loaded NPs eightfold in U87MG and HUVEC cells [98]. Furthermore, the combination of RGD and folate (FA) demonstrates the preferential accumulation of PTX–hydrogels [99] and PTX–mesoporous silica nanoparticles (MSNs) [100] at tumor sites, enhancing anti-tumor efficacy while reducing adverse effects. Additionally, the deubiquitinase OTUD5 has been shown to reduce paclitaxel sensitivity, whereas XBP1s activation can enhance it [101,102]. Combining RGD with glucose or fructose has also proven effective, maximizing the accumulation of PTX–liposomes at tumor sites compared to free PTX, uncoated liposomes, singly functionalized liposomes, and co-functionalized liposomes created through physical blending [103,104]. Novel peptide ligands such as PLZ4 (_c_[CQDGRMGFC]) and OA02 (cdG-HoCit-GPQc-Ebes-K-alkyne) have shown promise in selectively targeting ovarian and bladder cancer cells, respectively. These ligands have led to favorable anticancer efficacy of PTX–NPs in patient-derived bladder cancer xenografts and SKOV3 ovarian cancer xenografts [41,105]. Additionally, peptides like H2009.1 (DALRLQGTLR) exhibit high affinity for α_v_β_6_ integrin. The tetrameric H2009.1–PTX conjugate has demonstrated selective cytotoxicity and robust anticancer efficacy against non-small cell lung cancer (NSCLC) (Figure 4b) [40]. However, it is worth noting that some patients with lung squamous cell carcinoma may respond poorly to nab–paclitaxel therapy due to differences in metabolic profiles [106].

#### 3.1.3. Neuropilin-1

Neuropilin-1 (NRP-1) is highly expressed in ovarian, breast, prostate, and pancreatic cancers [107,108,109]. The NGR peptide, a ligand designed to target NRP-1, has shown promise in enhancing cellular uptake, prolonging systemic circulation, and increasing the tumor-suppressive effects of delivered paclitaxel (PTX) [110]. Molecular amphiphilicity promotes self-assembly [111,112], and the iNGR peptide (_c_[CRNGRGPDC]), composed of the NGR and CendR motifs, significantly improves tumor penetration compared to linear NGR peptides. Micelles incorporating iNGR, PEG, and PTX (iNGR-PEG-PTX8/PTX) exhibit remarkable tumor accumulation and enhanced survival rates in TNBC-bearing mice, outperforming conventional treatments like Taxol and non-targeted nanoparticles (NPs) [57]. Further advancements have been achieved with peptides such as CK3 (CLKADKAKC), RGE (RGERPPR), tLyP-1 (CGNKRTR), and A7R (ATWLPPR)-cysteine, each exhibiting unique interactions with NRP receptors. For instance, CK3-functionalized PEGylated poly(D, L-lactide) PTX-loaded micelles (CK3-PM-PTX) show a 2.2-fold enhancement in cell penetration compared to non-targeted NPs [59]. Similarly, RGE-grafted PEGylated-PLGA–paclitaxel nanoparticles (RGE-PEG/PLGA-PTX NPs) demonstrate increased cellular uptake in U87 MG and HUVEC cells [113]. A7R-cysteine peptide-cloaked PTX–liposomes (A7RC-LIPs) preferentially target delivery to MDA-MB-231 cells [114]. Furthermore, a combination of A7R and RGD co-modified liposomes encapsulating PTX significantly enhances cellular uptake in A549 and HUVEC cells [115].

The peptide tLyP-1 has emerged as a novel ligand for NRP receptors. Karmali et al. revealed that tLyP-1 effectively delivers PTX to extravascular tumor sites [62]. A combination of tLyP-1 and FA demonstrates the selective tumor-targeting ability of loaded PTX without undesired side effects [116]. However, studies exploring peptide modifications highlight the challenges of using D-peptide isomers. For example, while Lc(LyP-1) specifically binds to the p32/gC1qR protein, which is highly expressed on cancer cell membranes, the D-conformational peptide Dc(LyP-1)-functionalized micelles exhibit increased serum stability but reduced efficiency in targeting brain metastatic tumors [117].

#### 3.1.4. Epidermal Growth Factor Receptor (EGFR)

The epidermal growth factor receptor (EGFR) is highly expressed in breast, head and neck, non-small cell lung, and prostate cancers [118]. An anti-EGFR antibody–drug conjugate has demonstrated resistance to HER2-targeted drugs [119]. EGFR-targeted nanoparticles (EGFR-p; YHWYGYTPQNVI-GGGSGGGSC-modified NPs) have shown remarkable improvements in PTX delivery efficacy [45]. Additionally, a hexapeptide, LT6 (LARLLT), has been identified as an EGFR mimic. DOX/PTX-loaded LT6-coated micelles demonstrated significantly improved efficacy, with 4.8-fold lower IC50 values compared to unmodified micelles and 18.2-fold lower IC50 values compared to free DOX/PTX against SKOV3 cells [47].

EGFR type 2 (VEGFR2 or KDR) is specifically overexpressed in ovarian cancer [120], colorectal cancer [121], breast cancer [122], etc. The peptide K237 (HTMYYHHYQHHL) has exhibited a strong binding affinity to KDR [75]. K237-modified PTX-loaded nanoparticles (K237-PTX-NPs) have demonstrated pronounced antiangiogenic effects in HCT-15 and HUVEC cells [123,124]. Similarly, the application of a small angiogenesis-homing peptide, APRPG, for VEGFR-mediated targeting has driven significant advancements. Functionalizing PTX-encapsulated PEG-PLGA micelles with APRPG (APRPG-PEG-Mic) enhanced cellular penetration, tumor tissue accumulation, and prolonged tumor growth inhibition [74]. Moreover, the heptapeptide A7R has shown a high affinity for both VEGFR2 and NRP-1. Glycosylated A7R-tethered PTX-loaded NPs have exhibited superior anti-cancer efficacy in glioma xenograft models, demonstrating enhanced therapeutic potency and efficiency [61,125].

#### 3.1.5. Transferrin Receptor (TfR)

The transferrin receptor (TfR) is overexpressed in a variety of cancers, including breast, liver, brain, lung, ovarian, thyroid, esophageal, and colon cancers [126]. The TfR-targeting peptide T12(THRPPMWSPVWP) functionalized on PTX-loaded PEG-PLA micelles (TfR-T12-PMs) has significantly inhibited the proliferation of U87MG cells in vitro. These micelles have also demonstrated promising anti-glioma effects and extended survival rates in vivo [69]. Similarly, the heptapeptide T7 (HAIYPRH), particularly in its D-form DT7 (haiyprh), exhibited enhanced binding affinity to TfR. The combination of magnetic guidance with T7 increased the brain delivery of PTX-loaded NPs fivefold compared to unmodified NPs [70]. Additionally, the D-form DT7 peptide showed potent anti-proliferative effects when used to deliver PTX and cediranib in vivo [127].

#### 3.1.6. Somatostatin Receptor Type 2 (SSTR2)

**Figure 5:**
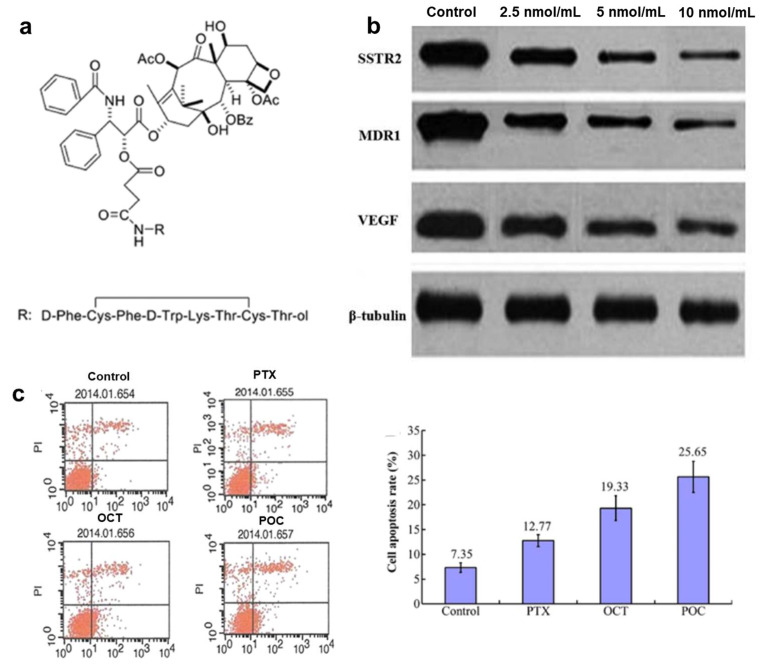
(**a**) The structure of paclitaxel–octreotide conjugate (POC). (**b**) Impact of POC on the protein expression of SSTR2 and MDR1 in A2780/Taxol cells. (**c**) The levels of cell apoptosis exhibited a pattern as observed in the control group [131].

Somatostatin receptor type 2 (SSTR2) is highly prevalent in glioma cells and the endothelial cells of proliferating vessels within gliomas [128]. Tyr-3-octreotide (TOC, F_c_[CYWK-^ξ^Thr-C]-^ξ^Thr-ol) is a well-established specific ligand for SSTR2. Increasing evidence suggests that octreotide–paclitaxel conjugates impede Calu-6 and A549 proliferation through a concentration- and time-dependent mechanism [129], offering a highly targeted chemotherapeutic approach [130]. Furthermore, treatment with the paclitaxel–octreotide conjugate (POC) resulted in higher levels of cell apoptosis than treatment with either paclitaxel or octreotide (OCT) alone. Additionally, the expression of multidrug resistance 1 (MDR1) and vascular endothelial growth factor (VEGF) at both the mRNA and protein levels decreased in a dose-dependent fashion (Figure 5). Moreover, TOC coating PTX-loaded NPs (TOC-PSM) significantly extended the tumor accumulation, enhanced the bio-distribution, and improved the antiangiogenic effects compared to Taxol and unmodified NPs [66]. As anticipated, TOC–PTX conjugates primarily interacted with SSTRs, prompting cancer cell apoptosis with reduced cytotoxicity towards Chinese hamster ovary cells compared to free PTX.

Subsequent investigations revealed that lanreotide exhibited a twofold increase in affinity for SSTR2 compared to octreotide [67]. Lanreotide-functionalized PTX-loaded PEG-b-PCL micelles (lanreotide-PM-PTX) demonstrated potent cytotoxicity and cellular uptake in SSTR2^+^ cancer cells in vitro, leading to enhanced tumor accumulation and inhibition in vivo [68].

#### 3.1.7. Acetylcholine Receptor (AChR)

**Figure 6:**
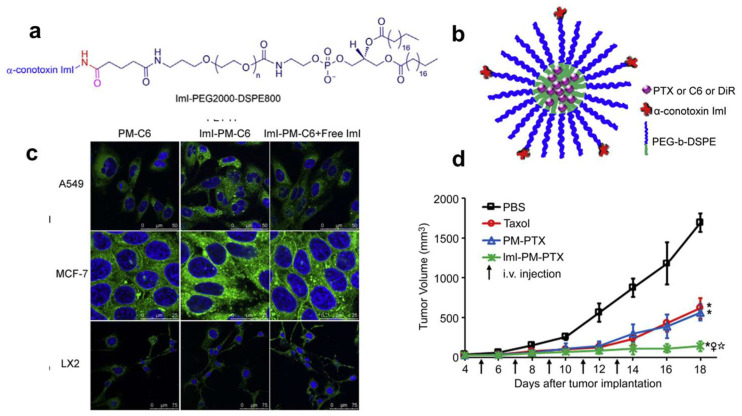
(**a**) The structure of ImI-PEG-DSPE copolymer. (**b**) Illustration of ImI-modified micelles (ImI-PMs). (**c**) Confocal microscopy images of A549, MCF-7, and LX2 cells following incubation with PM-C6 or ImI-PM-C6. (**d**) Tumor growth curves of mice treated with PBS, Taxol, PM-PTX, or ImI-PM-PTX. Each formulation was administered to the mice via the tail vein every other day for a total of 5 doses. The PTX dosage was 10 mg/kg. * *p* < 0.05, vs. PBS; ** *p* < 0.01, vs. PBS; ♀ *p* < 0.05, vs. Taxol; ☆ *p* < 0.05, vs. PM-PTX [30].

The acetylcholine receptor (AChR) is highly expressed in glioblastomas [132]. The rabies virus glycoprotein (RVG, YTIWMPENPRPGTPCDIFTNSRGKRASNGGGGC) peptide demonstrates highly specific binding with AChR, enabling RVG-conjugated nanoparticles carrying PTX (RVG-PTX-NPs) to exhibit sustained brain-targeting capabilities in human glioma-bearing mice [31]. In particular, the alpha7 nicotinic acetylcholine receptor (α7 nAChR) presents an appealing target due to its significant and specific role in tumors, influencing processes like migration, apoptosis, secretion, angiogenesis, and invasion [133]. ImI (G_c_[C_c_(CSDPRC]AWRC)-NH_2_), a member of the α-conotoxins, effectively inhibits α7 nAChR with a high affinity [134]. The ImI-PEG-DSPE copolymer self-assembled into micelles (ImI-PMs). As a result, paclitaxel-loaded ImI-PMs exhibited remarkable tumor-targeting effectiveness and enhanced antitumor efficacy in MCF-7 tumor-bearing nu/nu mice overexpressing α7 nAChR (Figure 6) [30].

#### 3.1.8. Luteinizing Hormone-Releasing Hormone Receptor (LHRH-R)

The luteinizing hormone-releasing hormone receptor (LHRH-R) is highly expressed in breast, prostate, endometrial, ovarian, bladder, pancreatic, colorectal, renal, and hepatic cancers, uveal melanoma, melanoma, and non-Hodgkin lymphoma [135]. Degarelix (Ac-^D^βNal-f(4-Cl)-γPal-S-F(4-S-dihydroorotamido)-f(4-ureido)-L-K(iPr)-P-a-NH_2_) served as a potent candidate due to its specific binding to LHRH-R. Wang et al. has investigated the potential of degarelix as a targeting agent for paclitaxel. The degarelix–PTX conjugate exhibited significantly enhanced serum stability (t_1/2_ > 10 h), demonstrating increased cytotoxicity against MCF-7 and HT-29 cells compared to 3T3 mouse embryonic fibroblast cells [55]. It was suggested that the LHRH peptide substantially enhanced tumor accumulation and inhibited tumor growth across all delivery nanoplatforms with minimal side effects [136]. The LHRH peptide-functionalized PTX-loaded PLGA-PEG copolymer displayed efficient targeting and transport capabilities [137]. To enhance the affinity further, a combination of the LHRH peptide and the AE105 peptide (D-Cha-FsrYLWS) was incorporated into iron oxide nanoparticles (IONPs) for PTX delivery. The approach showed a significant improvement in cytotoxicity compared to singly modified IONPs, resulting in a tenfold reduction in the effective dosage compared to free PTX [54].

#### 3.1.9. Gastrin-Releasing Peptide Receptor (GRPR)

The gastrin-releasing peptide receptor (GRPR) is highly expressed in breast, prostate, lung, and gastrointestinal cancers [138,139]. Bombesin (BN, PYR-QRLGNQWAVGHLM-NH_2_) shows a strong affinity for mammalian GRPR, providing targeting capabilities for breast cancer (GRPR positive). PLGA-based nanoparticles carrying BN-conjugated Lutetium-177 and PTX demonstrated controlled drug release dependent on pH reduction (pH = 7.4~5.3), resulting in an improved cellular uptake and cytotoxic effects [51]. The truncated form of bombesin, BBN(6_14) (YQWAVGHLM-NH_2_), displayed a sustained affinity for GRPR-positive human cancer cells, and BBN(6_14)–PTX conjugates exhibited significant cytotoxic effects with pronounced efficacy [52].

#### 3.1.10. Glucose-Regulated Protein 78 (GRP78)

Glucose-regulated protein 78 (GRP78) can be localized on the surfaces of specific cancer cells, such as glioma stem cells, glioma cells, and the endothelium of blood vessels [140]. Micelles designed for brain targeting of insoluble drugs can be effectively delivered via a nose-to-brain approach [141]. Studies have identified that _L_-VAP (SNTRVAP), _D_-VAP (sntrvap), and _RI_-VAP (pavrtns) exhibit a strong binding affinity to GRP78. Notably, _D_-VAP and _RI_-VAP demonstrate significantly greater tumor accumulation than _L_-VAP. Polymeric micelles containing PTX and _D_-VAP (or _RI_-VAP) show superior anticancer effects compared to Taxol, uncoated nanoparticles, and _L_-VAP modified nanoparticles. Additionally, _L_-peptide (RLLDTNRPLLPY) serves as another specific ligand for GRP78. The stability of drug applications plays a critical role [142,143]. Research indicates that L-peptide-coated PTX-loaded nanoparticles (L-CS-g-PNIPAM-PTX) exhibit enhanced specificity and anti-proliferative effects in MDA-MB-231 cells (GRP78+), leading to an extended median survival time in breast cancer-bearing mice with complete inhibition of tumor growth [49]. Furthermore, Passarella et al. revealed that GIRLRG-modified nanocomplexes increase the accumulation and induce apoptosis in irradiated breast cancers over a 3-week period, further underscoring the potential significance of targeting GRP78 in cancer therapies [50].

#### 3.1.11. Nucleolin (NCL)

**Figure 7:**
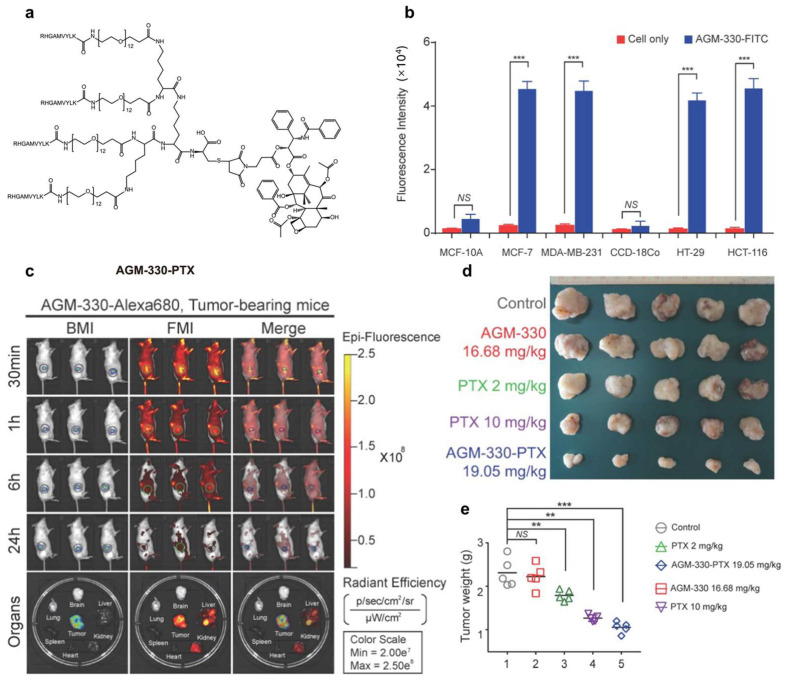
(**a**) The structure of AGM-330-PTX. (**b**) FACS analysis demonstrating the specificity of AGHM-330. (**c**) Fluorescence imaging of live mice at 30 min, 1 h, 6 h, and 24 h post-intravenous delivery of 10 nmol AGM-330-Alexa680 in MDA-MB-231-luc tumor-bearing mice. Primary tumor volume (**d**) and primary tumor weights (**e**) of MDA-MB-231-luc cells inoculated mice after treatment. Notes: NS indicates *p* > 0.05; ** indicates *p* < 0.01; *** indicates *p* < 0.001 [63].

The nucleolin (NCL) protein is highly expressed in ovarian, gastric, breast, liver, and non-small cell lung cancers, pancreatic ductal adenocarcinoma, and acute myeloid leukemia [144,145]. The degradation of nucleolin could inhibit cancer cell proliferation [146]. Nucleolin-targeted PTX formulations have found a widespread application in ovarian and breast cancers [144,147]. Kim et al. identified AGM-330 (RHGAMVYLK) as a peptide aptamer with specific interactions with nucleolin. AGM-330-PTX conjugates exhibited remarkable tumor growth inhibition in rat models of breast cancer compared to free PTX (Figure 7) [63]. Furthermore, a novel tumor-targeting peptide F3 (KDEPQRRSARLSAKPAPPKPEPKPKKAPAKKC) demonstrated a robust binding affinity for nucleolin. The effectiveness of F3-conjugated PEG-PLA nanocomplexes carrying PTX (F3-NP-PTX) was evident through their enhanced cellular uptake by MCF-7 cells, showcasing their superior antitumor activity both in vitro and in vivo compared to unmodified NPs [64].

### 3.2. Cell Adhesion Molecules and Extracellular Matrix Proteins Targeting Peptide Aptamers

#### 3.2.1. CD44

The CD44 receptor represents a widely targeted feature specific to tumors. Notably, a conspicuous elevation in CD44 isoforms (CD44_v3_ and CD44_v6_) was detected in diverse malignancies, including pancreatic, ovarian, and liver cancers [148]. The A5G27 peptide (Ac-KRLVSYNGIIFFLR) has exhibited a high affinity for CD44 isoforms. The targeted polymer–drug conjugate (P-(A5G27)-PTX) demonstrated an enhanced toxicity towards cancer cells that overexpress CD44 compared to the untargeted copolymer. In vivo studies have shown that a single intravenous dose of P-(A5G27)-PTX extended the survival of C57BL/6 mice with established B16-F10 lung metastases. Furthermore, when administered intraperitoneally to BALB/c mice bearing subcutaneous 4T1 tumors, P-(A5G27)-PTX significantly impeded the growth of primary tumors, increased median mouse survival, and reduced the number of 4T1 metastases in the lungs compared to the nontargeted copolymer [43].

#### 3.2.2. CD56

The neural cell adhesion molecule (NCAM), also known as CD56, is a cancer-associated antigen that is prominently expressed in different cancer types, such as glioblastoma, melanoma, neuroblastoma, and small cell lung cancer [149]. Subsequently, the NCAM-targeting peptide (NTP, GASKKPAANIKA) has been utilized as an innovative agonist with the potential for nanoparticle delivery, leading to the development of pharmacosomes for cancer treatment [150]. Research indicated that coating dendritic polyglycerol (PG)-conjugated PEG micelles with a PTX payload (PG-NTP-PTX-PEG) enhanced the cellular uptake and binding affinity to IMR-32 cells, leading to an increased inhibition of tumor migration and angiogenesis in vitro [56].

#### 3.2.3. Fibronectin Extra Domain B (EDB)

Research has revealed that fibronectin extra domain B (EDB) is significantly overexpressed on glioma cancer cells and neovascular endothelial cells. Gu et al. has investigated the effects of the APTEDB peptide (SSSPIQGSWTWENGKCWTWKGIIRLEQ) for delivering PTX, which exhibited a high specificity and binding affinity to EDB. The APTEDB-conjugated PTX-loaded PEG-PLA nanoparticles showed increased cellular uptake through caveolae and lipid raft-mediated endocytosis in an energy-dependent manner. Moreover, significant improvements in PTX-induced apoptosis and antiangiogenic effects were observed against U87MG cells in vitro, with a greater accumulation within the glioma and superior anti-tumor efficacy demonstrated in vivo [44].

#### 3.2.4. Heparan Sulfate

Heparan sulfate is known to be overexpressed in gliomas, and the CGKRK peptide has proven to be an effective ligand for targeting the glioma neo-vasculature while exhibiting minimal binding to normal vessels. Studies have shown that CGKRK peptide-conjugated PEG-PCL NPs (CGKRK-NP) are endocytosed by HUVEC cells via a caveolae-mediated pathway. These nanoparticles have demonstrated the capability to penetrate 3D tumor spheroids and tumors effectively. When loaded with PTX, they significantly reduced tumor volumes in U87MG-bearing mice [53]. Furthermore, redox-sensitive nanoparticles modified with the CGKRK peptide, co-encapsulating PTX and borneol, have displayed enhanced transport across the blood-brain barrier (BBB), increased tumor accumulation, and improved anti-glioma activity [151].

#### 3.2.5. Annexin A1

Annexin A1 is highly expressed in gastric cancer, breast cancer, and the vascular endothelial cells within solid tumors [152,153]. The combination of paclitaxel and minnelide has shown significant clinical efficacy in treating gastric cancer [154]. The IF-7 peptide (IFLLWQR) exhibits selective binding to annexin A1, enabling the effective penetration of cancer cells. Modified with the IF-7 motif, pH-responsive micelle frameworks were engineered into size-shrinkable nanoclusters (IF-7-MNC). The evaluation in 3D tumor spheroids demonstrated that the penetration capacity of IF-7-PMNC was 4.5 times higher than that of plain NPs (MNC) after a 24-h incubation period. Significantly, PTX-loaded IF-7-MNC (IF-7-PMNC) displayed potent tumor suppression, increasing the inhibitory rate to 89% compared to plain nanoparticles (PMNC) [32].

### 3.3. Immune Checkpoint Proteins and Intracellular Regulators Targeting Peptide Aptamers

#### 3.3.1. Programmed Cell Death Protein 1 (PD-1)

The process of identifying and validating targets can be effectively carried out using PROTAC technology [155,156]. The transmembrane protein programmed cell death protein 1 (PD-1) and its ligand (PD-L1) play a crucial role in cancer immunotherapy. PD-L1 expression is notably elevated in various cancer types, including ovarian, melanoma, and lung cancers. The dissociation constant (Kd) value of the D-PPA-1 peptide (nyskptdrqyhf) binding to PD-L1 was measured at 510 nM, demonstrating an exceptional resistance to degradation and blocking properties [65]. A dual-targeting CD peptide (comprising D-PPA-1 and CGKRK) was engineered for delivery systems, with D-mannose being able to target PD-1 for lysosomal degradation [157]. The PTX-loaded PCL nanoparticles (CD-NP-PTX) demonstrated a strong affinity for tumor cells and vascular endothelial cells, resulting in significant enhancements in cytotoxicity and the prevention of angiogenesis. The treatment notably extended median survival times and exhibited a clear synergistic effect in inhibiting the PD-1/PD-L1 pathway [46].

#### 3.3.2. Vav 3 Guanine Nucleotide Exchange Factor (VAV3)

Vav 3 guanine nucleotide exchange factor (VAV3) has been identified as upregulated in glioma-initiating cells, with its overexpression associated with crucial processes like apoptosis, metastasis, and tumor growth [158]. The heptapeptide glioma-initiating cell peptide (GICP, SSQPFWS) has shown a strong affinity for VAV3 receptors and human umbilical vein endothelial cells (HUVECs). By utilizing GICP, PLA micelles loaded with PTX (GICP-PEG-PLA) exhibited enhanced accumulation, penetration, and inhibition of tumor growth compared to non-targeted nanoparticles [73]. Additionally, quorum-sensing (QS) peptides have shown the ability to traverse the blood-brain barrier (BBB), with _L_-WSW (SYPGWSW) demonstrating brain clearance [159]. Stability is a key point for the clinical application of drugs [160]. To address the instability of _L_-WSW, researchers have developed its novel retro-inverso isomer, _D_-WSW, which exhibited increased antitumor efficacy. Micelles loaded with PTX encoded with _D_-WSW (DWSW Micelle/PTX) showed significant tumor accumulation, leading to a considerable extension of median survival time in vivo [161].

#### 3.3.3. Tax-Interacting Protein 1 (TIP-1)

Research has been conducted for exploring the potential applications of Tax-interacting protein 1 (TIP-1), which shows an elevated expression in human invasive breast cancer and the infiltrative growth of human glioblastoma [162,163]. Hariri and colleagues have identified a heptapeptide (HVGGSSV) with a high affinity for TIP-1, suggesting its utility in guiding nanogold for delivering PTX and CPT. These nanoparticles showed a 4-fold reduction in tumor growth compared to the untargeted group, primarily attributed to their significant accumulation in the tumor tissue [72].

Although a peptide aptamer is promising for tumor targeted therapy, the therapeutic use of peptides faces challenges due to their in vivo metabolic instability [164]. Peptides are highly vulnerable to degradation by various proteolytic enzymes because of the amido bonds in their sequences. There are over 550 proteases spread throughout the human body [165]. Even in the presence of just 10% human serum, linear peptides can degrade considerably within 24 h [166]. To enhance peptide stability, strategies such as protecting the N- and C-termini, replacing the L-amino acids with their D-forms, modifying the amino acids, cyclizing, using nanoparticle formulations, and increasing the molecular size are currently utilized [164,167]. The challenges related to peptide stability may be overcome in the future with the promising development of new technologies and methods.

## 4. Conclusions

Paclitaxel, a potent chemotherapeutic agent widely used in cancer treatment, has demonstrated significant efficacy in inducing cell death and inhibiting cell division. However, its systemic toxicity limits its dosage and effectiveness. The development of peptide aptamer–paclitaxel conjugates represents an innovative strategy in cancer therapy, combining the tumor-specific targeting capability of peptide aptamers with the cytotoxic potency of paclitaxel. Moreover, the peptide aptamer notably improves the solubility of paclitaxel, eliminating the need for the toxic Cremophor in Taxol. As a result, the method holds great promise for advancing oncology by offering a more selective and less toxic treatment option for cancer patients.

However, challenges such as the stability, immunogenicity, and pharmacokinetics of peptide aptamers need to be addressed before translating into clinical practice. Additionally, certain receptors highly expressed in tumors may also be present in specific normal tissues, raising concerns about their off-target effects and toxicity. A careful evaluation of the peptide aptamer specificity and the safety profile of the peptide aptamer–paclitaxel conjugates (PAPCs) is crucial to mitigate these risks. Current research efforts are focused on optimizing the peptide sequences, improving the drug–peptide conjugate designs, and exploring novel targeting strategies using advanced artificial intelligence techniques [168]. With continued progress in these areas, peptide aptamer-based therapies have the potential to become a standard of care for various cancer types, offering patients improved therapeutic outcomes and reduced toxicity.

## Figures and Tables

**Table 1:** Peptide aptamers utilized in PTX-based anticancer strategies.

Target	High Expression ^1^	Peptide Ligand	Sequence	Phase	Ref ^2^
LRP-1	Glioma cancer	Angiopep-2	TFFYGGSRGKRNNFKTEEY	Clinical stage III	[28]
		RAP12	EAKIEKHNHYQK	Preclinical	[29]
AChR	Glioblastomas	ImI	G_c_[C_c_(CSDPRC]AWRC)-NH_2_	Preclinical	[30]
		RVG	YTIWMPENPRPGTPCDIFTNSRGKRASNGGGGC	Preclinical	[31]
Annexin A1	Gastric and breast cancers	IF-7	IFLLWQR	Preclinical	[32]
α_v_β_3_ integrin α-3 integrin	Osteosarcomas, neuroblastomas, glioblastomas, malignant melanomas, breast, lung, and prostate cancers	iRGD	_c_[CRGDKGPDC]	Preclinical	[33,34,35]
		RGD	RGD/_c_[RGD]	Preclinical	[36,37,38]
		c(RGDfK)	_c_[RGDfK]	Preclinical	[39]
		PLZ4	_c_[CQDGRMGFC]	Preclinical	[40]
		OA02	cdG-HoCit-GPQc-Ebes-K-alkyne	Preclinical	[41]
α_v_β_6_ integrin		H2009.1	DALRLQGTLR	Preclinical	[42]
CD44 isoforms	Pancreatic, ovarian, and liver cancers	A5G27	Ac-KRLVSYNGIIFFLR	Preclinical	[43]
EDB	Glioma cancer	APTEDB	SSSPIQGSWTWENGKCWTWKGIIRLEQ	Preclinical	[44]
EGFR	Breast, head and neck, non-small cell lung, and prostate cancers	EGFR-p	YHWYGYTPQNVI-GGGSGGGSC	Preclinical	[45]
		AEYLR	AEYLR	Preclinical	[46]
		LT6	LARLLT	Preclinical	[47]
GRP78	Glioma cancer	_L_-VAP	SNTRVAP	Preclinical	[48]
		_D_-VAP	sntrvap	Preclinical	
		_RI_-VAP	pavrtns	Preclinical	
		_L_-peptide	RLLDTNRPLLPY	Preclinical	[49]
		GIRLRG	GIRLRG	Preclinical	[50]
GRPR	Breast, prostate, lung, and gastrointestinal cancers	BN	PYR-QRLGNQWAVGHLM-NH_2_	Preclinical	[51]
		BBN(6_14)	YQWAVGHLM-NH_2_	Preclinical	[52]
Heparan sulfate	Glioma cancer	CGKRK	CGKRK	Preclinical	[53]
LHRH-R	Breast, prostate, endometrial, ovarian, bladder, pancreatic, colorectal, renal, and hepatic cancers, uveal melanoma, melanoma, and non-Hodgkin lymphoma	AE105	D-Cha-FsrYLWS	Preclinical	[54]
		Degarelix	Ac-^D^βNal-f(4-Cl)-γPal-S-F(4-S-dihydroorotamido)-f(4-ureido)-L-K(iPr)-P-a-NH_2_	Preclinical	[55]
NCAM	Glioblastoma, melanoma, neuroblastoma, and small cell lung cancers	NTP	GASKKPAANIKA	Preclinical	[56]
NRP-1	Ovarian, breast, prostate, and pancreatic cancers	iNGR	_c_[CRNGRGPDC]	Preclinical	[57,58]
		CK3	CLKADKAKC	Preclinical	[59]
		RGE	RGERPPR	Preclinical	[60]
		A7R	ATWLPPR	Preclinical	[61]
		tLyP-1	CGNKRTR	Preclinical	[62]
NCL	Ovarian, gastric, breast, liver and non-small cell lung cancers, pancreatic ductal adeno-carcinoma, and acute myeloid leukemia	AGM-330	RHGAMVYLK	Preclinical	[63]
		F3	KDEPQRRSARLSAKPAPPKPEPKPKKAPAKKC	Preclinical	[64]
PD-L1	Ovarian, melanoma, and lung cancers	_D-_PPA-1	nyskptdrqyhf	Preclinical	[65]
SSTR2	Glioma cancer	Tyr-3-octreotide	F _c_[CYWK-^ξ^Thr-C]-^ξ^Thr-ol	Preclinical	[66]
		lanreotide	βNal-_c_[CVKWYC]T-NH_2_	Preclinical	[67,68]
TfR	Breast, liver, brain, lung, ovarian, thyroid, esophageal, and colon cancers	TfR-T12	THRPPMWSPVWP	Preclinical	[69]
		T7	HAIYPRH	Preclinical	[70]
		DT7	haiyprh	Preclinical	[71]
TIP-1	Breast cancer and glioblastoma	HVGGSSV	HVGGSSV	Preclinical	[72]
VAV3	Glioma cancer	GICP	SSQPFWS	Preclinical	[73]
		WSW	SYPGWSW	Preclinical	[48]
VEGFR2	Ovarian, colorectal, and breast cancers	APRPG	APRPG	Preclinical	[74]
		K237	HTMYYHHYQHHL	Preclinical	[75]
		A7R	ATWLPPR	Preclinical	[76]

^1^ In cancers. ^2^ Ref, Reference; Uppercase letter, L-configuration amino acid; Lowercase letter, D-configuration amino acid; c, cyclo; βNal, 2-naphthyl; ^D^βNal, D-configuration 2-naphthyl; γPal, 3-pyridin-3-ylpropanoyl; ^ξ^Thr, DL-threonyl; Thr-ol, DL-threoninol; NH_2_, amidation; Ac, acetylation; Cha, cyclohexyl-(L)-alanine.

## Data Availability

The authors declare that the source data are provided in the original paper.

## References

[1] Anand U., Dey A., Chandel A.K.S., Sanyal R., Mishra A., Pandey D.K., De Falco V., Upadhyay A., Kandimalla R., Chaudhary A. (2023). Cancer chemotherapy and beyond: Current status, drug candidates, associated risks and progress in targeted therapeutics. Genes Dis..

[2] Ma Y., Zhang H., Shen X., Yang X., Deng Y., Tian Y., Chen Z., Pan Y., Luo H., Zhong C. (2024). Aptamer Functionalized Hypoxia-potentiating Agent and Hypoxia-inducible Factor Inhibitor Combined with Hypoxia-activated Prodrug for Enhanced Tumor Therapy. Cancer Lett..

[3] Dai H., Abdullah R., Wu X., Li F., Ma Y., Lu A., Zhang G. (2022). Pancreatic cancer: Nucleic acid drug discovery and targeted therapy. Front. Cell Dev. Biol..

[4] Falah M., Rayan M., Rayan A. (2019). A Novel Paclitaxel Conjugate with Higher Efficiency and Lower Toxicity: A New Drug Candidate for Cancer Treatment. Int. J. Mol. Sci..

[5] Rayan M., Shadafny S., Falah A., Falah M., Abu-Lafi S., Asli S., Rayan A. (2022). A Novel Docetaxel-Biotin Chemical Conjugate for Prostate Cancer Treatment. Molecules.

[6] Zhao Q.G., Zhou Y.J., Cao D.X., Tang A.N., Kong D.M. (2023). DNA-Functionalized Porphyrinic Metal-Organic Framework-Based Drug Delivery System for Targeted Bimodal Cancer Therapy. J. Med. Chem..

[7] Wang Z., Liu Z., Qu J., Sun Y., Zhou W. (2024). Role of natural products in tumor therapy from basic research and clinical perspectives. Acta Mater. Medica.

[8] Ma Y., Yu S., Ni S., Zhang B., Kung A.C.F., Gao J., Lu A., Zhang G. (2021). Targeting strategies for enhancing paclitaxel specificity in chemotherapy. Front. Cell Dev. Biol..

[9] Liu Y., Lu X., Chen M., Wei Z., Peng G., Yang J., Tang C., Yu P. (2024). Advances in screening, synthesis, modification, and biomedical applications of peptides and peptide aptamers. Biofactors.

[10] Wang M., Liu J., Xia M., Yin L., Zhang L., Liu X., Cheng Y. (2024). Peptide-drug conjugates: A new paradigm for targeted cancer therapy. Eur. J. Med. Chem..

[11] Guo J., Zhang H., Lin W., Lu L., Su J., Chen X. (2023). Signaling pathways and targeted therapies for psoriasis. Signal Transduct. Target. Ther..

[12] Baghy K., Ladanyi A., Reszegi A., Kovalszky I. (2023). Insights into the Tumor Microenvironment-Components, Functions and Therapeutics. Int. J. Mol. Sci..

[13] van Weverwijk A., de Visser K.E. (2023). Mechanisms driving the immunoregulatory function of cancer cells. Nat. Rev. Cancer.

[14] Alas M., Saghaeidehkordi A., Kaur K. (2021). Peptide-Drug Conjugates with Different Linkers for Cancer Therapy. J. Med. Chem..

[15] Torrini F., Scarano S., Palladino P., Minunni M. (2023). Advances and perspectives in the analytical technology for small peptide hormones analysis: A glimpse to gonadorelin. J. Pharm. Biomed. Anal..

[16] Pan Y., Wang Q., Ma Y. (2024). Small RNAs in Cancer Therapy. Interdisciplinary Cancer Research.

[17] Amu G., Ma Y., Yu S., Zhang H., Chen Z., Ni S., Abdullah R., Xiao H., Zhang Y., Dai H. (2024). Unique quinoline orientations shape the modified aptamer to sclerostin for enhanced binding affinity and bone anabolic potential. Mol. Ther. Nucleic Acids.

[18] Amu G., Zhang G., Jing N., Ma Y. (2024). Developing Stapled Aptamers with a Constrained Conformation for Osteogenesis Imperfect Therapeutics. J. Med. Chem..

[19] Zhang H., Yu S., Ni S., Gubu A., Ma Y., Zhang Y., Li H., Wang Y., Wang L., Zhang Z. (2023). A bimolecular modification strategy for developing long-lasting bone anabolic aptamer. Mol. Ther. Nucleic Acids.

[20] Ma Y., Zhang Y., Chen Z., Tian Y., Zhang G., Mithun R. (2023). The Modification Strategies for Enhancing the Metabolic Stabilities and Pharmacokinetics of Aptamer Drug Candidates. Drug Metabolism and Pharmacokinetics.

[21] Masuda R., Phyu Thant K.P., Kawahara K., Oki H., Kadonosono T., Kobayashi Y., Koide T. (2024). A yeast two-hybrid system to obtain triple-helical ligands from combinatorial random peptide libraries. J. Biol. Chem..

[22] Kunamneni A., Ogaugwu C., Bradfute S., Durvasula R. (2020). Ribosome Display Technology: Applications in Disease Diagnosis and Control. Antibodies.

[23] Jaroszewicz W., Morcinek-Orlowska J., Pierzynowska K., Gaffke L., Wegrzyn G. (2022). Phage display and other peptide display technologies. FEMS Microbiol. Rev..

[24] Wu Y., Bertran M.T., Joshi D., Maslen S.L., Hurd C., Walport L.J. (2023). Identification of photocrosslinking peptide ligands by mRNA display. Commun. Chem..

[25] Li C., Hua Y., Pan D., Qi L., Xiao C., Xiong Y., Lu W., Dang Y., Gao X., Zhao Y. (2023). A rapid selection strategy for umami peptide screening based on machine learning and molecular docking. Food Chem..

[26] Ma Y., Yu Y., Zhang B., Lu A., Zhang G. (2023). Editorial: Aptamer-based structural biology, computational modelling, translational research and drug discovery, Volume II. Front. Cell Dev. Biol..

[27] Chen R., Liu E., Fang Y., Gao N., Zhang M., Zhang X., Chen W., Liang C., Zhang Y., Huang Y. (2024). Naturally sourced amphiphilic peptides as paclitaxel vehicles for breast cancer treatment. Biomater. Adv..

[28] Regina A., Demeule M., Che C., Lavallee I., Poirier J., Gabathuler R., Beliveau R., Castaigne J.P. (2008). Antitumour activity of ANG1005, a conjugate between paclitaxel and the new brain delivery vector Angiopep-2. Br. J. Pharmacol..

[29] Ruan H., Chai Z., Shen Q., Chen X., Su B., Xie C., Zhan C., Yao S., Wang H., Zhang M. (2018). A novel peptide ligand RAP12 of LRP1 for glioma targeted drug delivery. J. Control. Release.

[30] Mei D., Lin Z., Fu J., He B., Gao W., Ma L., Dai W., Zhang H., Wang X., Wang J. (2015). The use of α-conotoxin ImI to actualize the targeted delivery of paclitaxel micelles to α7 nAChR-overexpressing breast cancer. Biomaterials.

[31] Zou L., Tao Y., Payne G., Do L., Thomas T., Rodriguez J., Dou H. (2017). Targeted delivery of nano-PTX to the brain tumor-associated macrophages. Oncotarget.

[32] Chen Q., Liang H., Sun Y., Chen Y., He W., Fang X., Sha X., Li J. (2019). A carbohydrate mimetic peptide modified size-shrinkable micelle nanocluster for anti-tumor targeting and penetrating drug delivery. Int. J. Nanomed..

[33] Zhong Y., Su T., Shi Q., Feng Y., Tao Z., Huang Q., Li L., Hu L., Li S., Tan H. (2019). Co-administration of iRGD enhances tumor-targeted delivery and anti-tumor effects of paclitaxel-loaded PLGA nanoparticles for colorectal cancer treatment. Int. J. Nanomed..

[34] Li L., Yang M., Li R., Hu J., Yu L., Qian X. (2021). iRGD co-administration with paclitaxel-loaded PLGA nanoparticles enhance targeting and antitumor effect in colorectal cancer treatment. Anti-Cancer Agents Med. Chem..

[35] Hu H., Wang B., Lai C., Xu X., Zhen Z., Zhou H., Xu D. (2019). iRGD-paclitaxel conjugate nanoparticles for targeted paclitaxel delivery. Drug Dev. Res..

[36] Wang G., Wang Z., Li C., Duan G., Wang K., Li Q., Tao T. (2018). RGD peptide-modified, paclitaxel prodrug-based, dual-drugs loaded, and redox-sensitive lipid-polymer nanoparticles for the enhanced lung cancer therapy. Biomed. Pharmacother..

[37] Shi J., Liu S., Yu Y., He C., Tan L., Shen Y.M. (2019). RGD peptide-decorated micelles assembled from polymer-paclitaxel conjugates towards gastric cancer therapy. Colloids Surf. B Biointerfaces.

[38] Huang Z.G., Lv F.M., Wang J., Ca S.J., Liu Z.P., Liu Y., Lu W.Y. (2019). RGD-modified PEGylated paclitaxel nanocrystals with enhanced stability and tumor-targeting capability. Int. J. Pharm..

[39] Zhang P., Hu L., Yin Q., Feng L., Li Y. (2012). Transferrin-modified c[RGDfK]-paclitaxel loaded hybrid micelle for sequential blood-brain barrier penetration and glioma targeting therapy. Mol. Pharm..

[40] Pan A., Zhang H., Li Y., Lin T.Y., Wang F., Lee J., Cheng M., Dall’Era M., Li T., deVere White R. (2016). Disulfide-crosslinked nanomicelles confer cancer-specific drug delivery and improve efficacy of paclitaxel in bladder cancer. Nanotechnology.

[41] Xiao K., Li Y., Lee J.S., Gonik A.M., Dong T., Fung G., Sanchez E., Xing L., Cheng H.R., Luo J. (2012). “OA02” peptide facilitates the precise targeting of paclitaxel-loaded micellar nanoparticles to ovarian cancer in vivo. Cancer Res..

[42] Li S., Gray B.P., McGuire M.J., Brown K.C. (2011). Synthesis and biological evaluation of a peptide-paclitaxel conjugate which targets the integrin alphavbeta(6). Bioorganic Med. Chem..

[43] Zaiden M., Rütter M., Shpirt L., Ventura Y., Feinshtein V., David A. (2018). CD44-Targeted Polymer–Paclitaxel Conjugates to Control the Spread and Growth of Metastatic Tumors. Mol. Pharm..

[44] Gu G., Hu Q., Feng X., Gao X., Menglin J., Kang T., Jiang D., Song Q., Chen H., Chen J. (2014). PEG-PLA nanoparticles modified with APTEDB peptide for enhanced anti-angiogenic and anti-glioma therapy. Biomaterials.

[45] Lin Z.L., Ding J., Sun G.P., Li D., He S.S., Liang X.F., Huang X.R., Xie J. (2020). Application of paclitaxel-loaded EGFR peptide-conjugated magnetic polymeric liposomes for liver cancer therapy. Curr. Med. Sci..

[46] Sun Z., Zhang Y., Cao D., Wang X., Yan X., Li H., Huang L., Qu X., Kong C., Qin H. (2018). PD-1/PD-L1 pathway and angiogenesis dual recognizable nanoparticles for enhancing chemotherapy of malignant cancer. Drug Deliv..

[47] Lin W.J., Kao L.T. (2014). Cytotoxic enhancement of hexapeptide-conjugated micelles in EGFR high-expressed cancer cells. Expert Opin. Drug Deliv..

[48] Ran D., Mao J., Shen Q., Xie C., Zhan C., Wang R., Lu W. (2017). GRP78 enabled micelle-based glioma targeted drug delivery. J. Control. Release.

[49] Niu S., Bremner D.H., Wu J., Wu J., Wang H., Li H., Qian Q., Zheng H., Zhu L. (2018). l-peptide functionalized dual-responsive nanoparticles for controlled paclitaxel release and enhanced apoptosis in breast cancer cells. Drug Deliv..

[50] Passarella R.J., Spratt D.E., van der Ende A.E., Phillips J.G., Wu H., Sathiyakumar V., Zhou L., Hallahan D.E., Harth E., Diaz R. (2010). Targeted nanoparticles that deliver a sustained, specific release of Paclitaxel to irradiated tumors. Cancer Res..

[51] Gibbens-Bandala B., Morales-Avila E., Ferro-Flores G., Santos-Cuevas C., Melendez-Alafort L., Trujillo-Nolasco M., Ocampo-Garcia B. (2019). (177)Lu-Bombesin-PLGA (paclitaxel): A targeted controlled-release nanomedicine for bimodal therapy of breast cancer. Mater. Sci. Eng. C Mater. Biol. Appl..

[52] Safavy A., Raisch K.P., Matusiak D., Bhatnagar S., Helson L. (2006). Single-drug multiligand conjugates: Synthesis and preliminary cytotoxicity evaluation of a paclitaxel-dipeptide “scorpion” molecule. Bioconjugate Chem..

[53] Hu Q., Gao X., Kang T., Feng X., Jiang D., Tu Y., Song Q., Yao L., Jiang X., Chen H. (2013). CGKRK-modified nanoparticles for dual-targeting drug delivery to tumor cells and angiogenic blood vessels. Biomaterials.

[54] Ahmed M.S.U., Salam A.B., Yates C., Willian K., Jaynes J., Turner T., Abdalla M.O. (2017). Double-receptor-targeting multifunctional iron oxide nanoparticles drug delivery system for the treatment and imaging of prostate cancer. Int. J. Nanomed..

[55] Wang C., Ma Y., Feng S., Liu K., Zhou N. (2015). Gonadotropin-releasing hormone receptor-targeted paclitaxel-degarelix conjugate: Synthesis and in vitro evaluation. J. Pept. Sci..

[56] Vossen L.I., Markovsky E., Eldar-Boock A., Tschiche H.R., Wedepohl S., Pisarevsky E., Satchi-Fainaro R., Calderon M. (2018). PEGylated dendritic polyglycerol conjugate targeting NCAM-expressing neuroblastoma: Limitations and challenges. Nanomed. Nanotechnol. Biol. Med..

[57] Zhang Y., Lu Y., Zhang Y., He X., Chen Q., Liu L., Chen X., Ruan C., Sun T., Jiang C. (2017). Tumor-targeting micelles based on linear-dendritic PEG-PTX8 conjugate for triple negative breast cancer therapy. Mol. Pharm..

[58] Kang T., Gao X., Hu Q., Jiang D., Feng X., Zhang X., Song Q., Yao L., Huang M., Jiang X. (2014). iNGR-modified PEG-PLGA nanoparticles that recognize tumor vasculature and penetrate gliomas. Biomaterials.

[59] Wang Y., Zhao H., Peng J., Chen L., Tan L., Huang Y., Qian Z. (2016). Targeting therapy of neuropilin-1 receptors overexpressed breast cancer by paclitaxel-loaded CK3-conjugated polymeric micelles. J. Biomed. Nanotechnol..

[60] Ma Y., Dong Y., Li X., Wang F., Zhang Y. (2021). Tumor-Penetrating Peptide-Functionalized Ferritin Enhances Antitumor Activity of Paclitaxel. ACS Appl. Bio Mater..

[61] Wang H., Wang X., Xie C., Zhang M., Ruan H., Wang S., Jiang K., Wang F., Zhan C., Lu W. (2018). Nanodisk-based glioma-targeted drug delivery enabled by a stable glycopeptide. J. Control. Release.

[62] Karmali P.P., Kotamraju V.R., Kastantin M., Black M., Missirlis D., Tirrell M., Ruoslahti E. (2009). Targeting of albumin-embedded paclitaxel nanoparticles to tumors. Nanomed. Nanotechnol. Biol. Med..

[63] Kim J.H., Bae C., Kim M.J., Song I.H., Ryu J.H., Choi J.H., Lee C.J., Nam J.S., Kim J.I. (2020). A novel nucleolin-binding peptide for cancer theranostics. Theranostics.

[64] Cai Y., Xu Z.M., Shuai Q., Zhu F.T., Xu J., Gao X., Sun X.R. (2020). Tumor-targeting peptide functionalized PEG-PLA micelles for efficient drug delivery. Biomater. Sci..

[65] Chang H.N., Liu B.Y., Qi Y.K., Zhou Y., Chen Y.P., Pan K.M., Li W.W., Zhou X.M., Ma W.W., Fu C.Y. (2015). Blocking of the PD-1/PD-L1 interaction by a d-peptide antagonist for cancer immunotherapy. Angew. Chem..

[66] Banerjee I., De K., Mukherjee D., Dey G., Chattopadhyay S., Mukherjee M., Mandal M., Bandyopadhyay A.K., Gupta A., Ganguly S. (2016). Paclitaxel-loaded solid lipid nanoparticles modified with Tyr-3-octreotide for enhanced anti-angiogenic and anti-glioma therapy. Acta Biomater..

[67] Patel Y.C. (1999). Somatostatin and its receptor family. Front. Neuroendocrinol..

[68] Zheng N., Dai W., Du W., Zhang H., Lei L., Zhang H., Wang X., Wang J., Zhang X., Gao J. (2012). A novel lanreotide-encoded micelle system targets paclitaxel to the tumors with overexpression of somatostatin receptors. Mol. Pharm..

[69] Sun P., Xiao Y., Di Q., Ma W., Ma X., Wang Q., Chen W. (2020). Transferrin receptor-targeted PEG-PLA polymeric micelles for chemotherapy against glioblastoma multiforme. Int. J. Nanomed..

[70] Cui Y., Zhang M., Zeng F., Jin H., Xu Q., Huang Y. (2016). Dual-targeting magnetic PLGA nanoparticles for codelivery of paclitaxel and curcumin for brain tumor therapy. ACS Appl. Mater. Interfaces.

[71] Zhang H., Wu T., Yu W., Ruan S., He Q., Gao H. (2018). Ligand Size and Conformation Affect the Behavior of Nanoparticles Coated with in Vitro and in Vivo Protein Corona. ACS Appl. Mater. Interfaces.

[72] Hariri G., Edwards A.D., Merrill T.B., Greenbaum J.M., van der Ende A.E., Harth E. (2014). Sequential targeted delivery of paclitaxel and camptothecin using a cross-linked “nanosponge” network for lung cancer chemotherapy. Mol. Pharm..

[73] Zhang M., Chen X., Ying M., Gao J., Zhan C., Lu W. (2017). Glioma-targeted drug delivery enabled by a multifunctional peptide. Bioconjug. Chem..

[74] Guo P., Song S., Li Z., Tian Y., Zheng J., Yang X., Pan W. (2015). In vitro and in vivo evaluation of APRPG-modified angiogenic vessel targeting micelles for anticancer therapy. Int. J. Pharm..

[75] Lei H.T., An P., Song S.M., Liu X.Y., He L.W., Wu J., Meng L., Liu M.S., Yang J.S., Shou C.C. (2002). A novel peptide isolated from a phage display library inhibits tumor growth and metastasis by blocking the binding of vascular endothelial growth factor to its kinase domain receptor. J. Biol. Chem..

[76] Wang L., Zhao C., Lu L., Jiang H., Wang F., Zhang X. (2023). Transcytosable Peptide-Paclitaxel Prodrug Nanoparticle for Targeted Treatment of Triple-Negative Breast Cancer. Int. J. Mol. Sci..

[77] Li Y., Zheng X., Gong M., Zhang J. (2016). Delivery of a peptide-drug conjugate targeting the blood brain barrier improved the efficacy of paclitaxel against glioma. Oncotarget.

[78] Maletinska L., Blakely E.A., Bjornstad K.A., Deen D.F., Knoff L.J., Forte T.M. (2000). Human glioblastoma cell lines: Levels of low-density lipoprotein receptor and low-density lipoprotein receptor-related protein. Cancer Res..

[79] Sarantopoulos J., Gabrail N.Y., Moulder S.L., Brenner A.J., Smith C.L., Bouchard D., Elian K., Lawrence B., Castaigne J., Kurzrock R. (2010). ANG1005: Results of a phase I study in patients with advanced solid tumors and brain metastases. J. Clin. Oncol..

[80] Kumthekar P., Tang S., Brenner A.J., Kesari S., Anders C.K., Carrillo J.A., Chalasani P., Kabos P., Ahluwalia M.S., Ibrahim N.K. (2016). OS7.2 A Phase II Study of ANG1005, a novel BBB/BCB Penetratant Taxane in Patients with Recurrent Brain Metastases and Leptomeningeal Carcinomatosis from Breast Cancer. Neuro-Oncology.

[81] Kumthekar P., Lawrence B., Iordanova V., Ibrahim N., Mazanet R. (2019). Abstract OT1-06-01: ANG1005 in leptomeningeal disease (ANGLeD) trial: A randomized, open-label, phase 3 study of ANG1005 compared with Physician’s Best Choice in HER2-negative breast cancer patients with newly diagnosed leptomeningeal carcinomatosis and previously treated brain metastases. Cancer Res..

[82] Drappatz J., Brenner A., Wong E.T., Eichler A., Schiff D., Groves M.D., Mikkelsen T., Rosenfeld S., Sarantopoulos J., Meyers C.A. (2013). Phase I study of GRN1005 in recurrent malignant glioma. Clin. Cancer Res..

[83] Kozma G.T., Shimizu T., Ishida T., Szebeni J. (2020). Anti-PEG antibodies: Properties, formation, testing and role in adverse immune reactions to PEGylated nano-biopharmaceuticals. Adv. Drug Deliv. Rev..

[84] Xin H.L., Jiang X.Y., Gu J.J., Sha X.Y., Chen L.C., Law K., Chen Y.Z., Wang X., Jiang Y., Fang X.L. (2011). Angiopep-conjugated poly(ethylene glycol)-co-poly(epsilon-caprolactone) nanoparticles as dual-targeting drug delivery system for brain glioma. Biomaterials.

[85] Xin H., Sha X., Jiang X., Zhang W., Chen L., Fang X. (2012). Anti-glioblastoma efficacy and safety of paclitaxel-loading angiopep-conjugated dual targeting PEG-PCL nanoparticles. Biomaterials.

[86] Pachane B.C., Selistre-de-Araujo H.S. (2024). The Role of alphavbeta3 Integrin in Cancer Therapy Resistance. Biomedicines.

[87] Debordeaux F., Chansel-Debordeaux L., Pinaquy J.-B., Fernandez P., Schulz J. (2018). What about αvβ3 integrins in molecular imaging in oncology?. Nucl. Med. Biol..

[88] Ganipineni L.P., Ucakar B., Joudiou N., Riva R., Jerome C., Gallez B., Danhier F., Preat V. (2019). Paclitaxel-loaded multifunctional nanoparticles for the targeted treatment of glioblastoma. J. Drug Target..

[89] Meng S., Su B., Li W., Ding Y., Tang L., Zhou W., Song Y., Caicun Z. (2011). Integrin-targeted paclitaxel nanoliposomes for tumor therapy. Med. Oncol..

[90] Rizvi S.F.A., Abbas N., Zhang H., Fang Q. (2023). Identification of a pH-Responsive Peptide-Paclitaxel Conjugate as a Novel Drug with Improved Therapeutic Potential. J. Med. Chem..

[91] Ren L., Chen S., Li H., Zhang Z., Ye C., Liu M., Zhou X. (2015). MRI-visible liposome nanovehicles for potential tumor-targeted delivery of multimodal therapies. Nanoscale.

[92] Huang S., Zhang Y., Wang L., Liu W., Xiao L., Lin Q., Gong T., Sun X., He Q., Zhang Z. (2020). Improved melanoma suppression with target-delivered TRAIL and paclitaxel by a multifunctional nanocarrier. J. Control. Release.

[93] Colombo R., Mingozzi M., Belvisi L., Arosio D., Piarulli U., Carenini N., Perego P., Zaffaroni N., De Cesare M., Castiglioni V. (2012). Synthesis and Biological Evaluation (in Vitro and in Vivo) of Cyclic Arginine–Glycine–Aspartate (RGD) Peptidomimetic–Paclitaxel Conjugates Targeting Integrin αVβ3. J. Med. Chem..

[94] Zhang J., Wang S., Deng Z., Li L., Tan G., Liu X., Zheng H., Yan F. (2018). Ultrasound-triggered drug delivery for breast tumor therapy through iRGD-targeted paclitaxel-loaded liposome-microbubble complexes. J. Biomed. Nanotechnol..

[95] Kang T., Li Y., Wang Y., Zhu J., Yang L., Huang Y., Xiong M., Liu J., Wang S., Huang M. (2019). Modular engineering of targeted dual-drug nanoassemblies for cancer chemoimmunotherapy. ACS Appl. Mater. Interfaces.

[96] Li Y., Chen M., Yao B., Lu X., Song B., Vasilatos S.N., Zhang X., Ren X., Yao C., Bian W. (2020). Dual pH/ROS-responsive nanoplatform with deep tumor penetration and self-amplified drug release for enhancing tumor chemotherapeutic efficacy. Small.

[97] Ma Y., Zhu Y., Wang C., Pan D., Liu S., Yang M., Xiao Z., Yang X., Zhao W., Zhou X. (2018). Annealing novel nucleobase-lipids with oligonucleotides or plasmid DNA based on H-bonding or π-π interaction: Assemblies and transfections. Biomaterials.

[98] Schleich N., Po C., Jacobs D., Ucakar B., Gallez B., Danhier F., Preat V. (2014). Comparison of active, passive and magnetic targeting to tumors of multifunctional paclitaxel/SPIO-loaded nanoparticles for tumor imaging and therapy. J. Control. Release.

[99] Shu C., Sabi-mouka E.M.B., Wang X., Ding L. (2017). Self-assembly hydrogels as multifunctional drug delivery of paclitaxel for synergistic tumour-targeting and biocompatibility in vitro and in vivo. J. Pharm. Pharmacol..

[100] Yan H., You Y., Li X., Liu L., Guo F., Zhang Q., Liu D., Tong Y., Ding S., Wang J. (2020). Preparation of RGD peptide/folate acid double-targeted mesoporous silica nanoparticles and its application in human breast cancer MCF-7 cells. Front. Pharmacol..

[101] Liu X., Ma Z., Jing X., Wang G., Zhao L., Zhao X., Zhang Y. (2024). The deubiquitinase OTUD5 stabilizes SLC7A11 to promote progression and reduce paclitaxel sensitivity in triple-negative breast cancer. Cancer Lett..

[102] Wang J., Fan P., Shen P., Fan C., Zhao P., Yao S., Dong K., Ling R., Chen S., Zhang J. (2024). XBP1s activates METTL3/METTL14 for ER-phagy and paclitaxel sensitivity regulation in breast cancer. Cancer Lett..

[103] Fu Q., Zhao Y., Yang Z., Yue Q., Xiao W., Chen Y., Yang Y., Guo L., Wu Y. (2019). Liposomes actively recognizing the glucose transporter GLUT(1) and integrin alpha(v) beta(3) for dual-targeting of glioma. Arch. Pharm..

[104] Pu Y., Zhang H., Peng Y., Fu Q., Yue Q., Zhao Y., Guo L., Wu Y. (2019). Dual-targeting liposomes with active recognition of GLUT5 and alphavbeta3 for triple-negative breast cancer. Eur. J. Med. Chem..

[105] Lin T.Y., Li Y., Liu Q., Chen J.L., Zhang H., Lac D., Zhang H., Ferrara K.W., Wachsmann-Hogiu S., Li T. (2016). Novel theranostic nanoporphyrins for photodynamic diagnosis and trimodal therapy for bladder cancer. Biomaterials.

[106] Cao P., Zhang Q., Wu S., Sullivan M.A., Huang Y., Gong W., Lv Y., Zhai X., Zhang Y. (2023). Baseline differences in metabolic profiles of patients with lung squamous cell carcinoma responding or not responding to treatment with nanoparticle albumin-bound paclitaxel (nab-paclitaxel). Acta Mater. Medica.

[107] Jiang H., Xi Q., Wang F., Sun Z., Huang Z., Qi L. (2015). Increased expression of neuropilin 1 is associated with epithelial ovarian carcinoma. Mol. Med. Rep..

[108] Feng G.K., Liu R.B., Zhang M.Q., Ye X.X., Zhong Q., Xia Y.F., Li M.Z., Wang J., Song E.W., Zhang X. (2014). SPECT and near-infrared fluorescence imaging of breast cancer with a neuropilin-1-targeting peptide. J. Control. Release.

[109] Gray M.J., Wey J.S., Belcheva A., McCarty M.F., Trevino J.G., Evans D.B., Ellis L.M., Gallick G.E. (2005). Neuropilin-1 suppresses tumorigenic properties in a human pancreatic adenocarcinoma cell line lacking neuropilin-1 coreceptors. Cancer Res..

[110] Yang Y., Xie X., Yang Y., Zhang H., Mei X. (2015). Photo-responsive and NGR-mediated multifunctional nanostructured lipid carrier for tumor-specific therapy. J. Pharm. Sci..

[111] Amu G., Zhang X., Lu A., Zhang B., Ma Y., Zhang G. (2023). Nucleic acid amphiphiles: Synthesis, properties and applications. Mol. Ther. Nucleic Acids.

[112] Chen Z., Luo H., Gubu A., Yu S., Zhang H., Dai H., Zhang Y., Zhang B., Ma Y., Lu A. (2023). Chemically modified aptamers for improving binding affinity to the target proteins via enhanced non-covalent bonding. Front. Cell Dev. Biol..

[113] Yu J., Sun L., Zhou J., Gao L., Nan L., Zhao S., Peng T., Han L., Wang J., Lu W. (2017). Self-assembled tumor-penetrating peptide-modified poly(l-gamma-glutamylglutamine)-paclitaxel nanoparticles based on hydrophobic interaction for the treatment of glioblastoma. Bioconjug. Chem..

[114] Cao J.Y., Wang R., Gao N., Li M.H., Tian X.Y., Yang W.L., Ruan Y., Zhou C.L., Wang G.T., Liu X.Y. (2015). A7RC peptide modified paclitaxel liposomes dually target breast cancer. Biomater. Sci..

[115] Meng S., Su B., Li W., Ding Y., Tang L., Zhou W., Song Y., Li H., Zhou C. (2010). Enhanced antitumor effect of novel dual-targeted paclitaxel liposomes. Nanotechnology.

[116] Wang W., Li M., Zhang Z., Cui C., Zhou J., Yin L., Lv H. (2017). Design, synthesis and evaluation of multi-functional tLyP-1-hyaluronic acid-paclitaxel conjugate endowed with broad anticancer scope. Carbohydr. Polym..

[117] Zhang X., Wang F., Shen Q., Xie C., Liu Y., Pan J., Lu W. (2018). Structure Reconstruction of LyP-1: (L)c(LyP-1) Coupling by Amide Bond Inspires the Brain Metastatic Tumor Targeted Drug Delivery. Mol. Pharm..

[118] Ciardiello F., Tortora G. (2003). Epidermal growth factor receptor (EGFR) as a target in cancer therapy: Understanding the role of receptor expression and other molecular determinants that could influence the response to anti-EGFR drugs. Eur. J. Cancer.

[119] Gandullo-Sanchez L., Pandiella A. (2023). An anti-EGFR antibody-drug conjugate overcomes resistance to HER2-targeted drugs. Cancer Lett..

[120] Spannuth W.A., Nick A.M., Jennings N.B., Armaiz-Pena G.N., Mangala L.S., Danes C.G., Lin Y.G., Merritt W.M., Thaker P.H., Kamat A.A. (2009). Functional significance of VEGFR-2 on ovarian cancer cells. Int. J. Cancer.

[121] Zhong M., Li N., Qiu X., Ye Y., Chen H., Hua J., Yin P., Zhuang G. (2020). TIPE regulates VEGFR2 expression and promotes angiogenesis in colorectal cancer. Int. J. Biol. Sci..

[122] Higgins K.J., Liu S., Abdelrahim M., Yoon K., Vanderlaag K., Porter W., Metz R.P., Safe S. (2006). Vascular Endothelial Growth Factor Receptor-2 Expression Is Induced by 17β-Estradiol in ZR-75 Breast Cancer Cells by Estrogen Receptor α/Sp Proteins. Endocrinology.

[123] Yu D.H., Lu Q., Xie J., Fang C., Chen H.Z. (2010). Peptide-conjugated biodegradable nanoparticles as a carrier to target paclitaxel to tumor neovasculature. Biomaterials.

[124] Bai F., Wang C., Lu Q., Zhao M., Ban F.Q., Yu D.H., Guan Y.Y., Luan X., Liu Y.R., Chen H.Z. (2013). Nanoparticle-mediated drug delivery to tumor neovasculature to combat P-gp expressing multidrug resistant cancer. Biomaterials.

[125] Wang H., Wang S., Wang R., Wang X., Jiang K., Xie C., Zhan C., Wang H., Lu W. (2019). Co-delivery of paclitaxel and melittin by glycopeptide-modified lipodisks for synergistic anti-glioma therapy. Nanoscale.

[126] Feng G., Arima Y., Midorikawa K., Kobayashi H., Oikawa S., Zhao W., Zhang Z., Takeuchi K., Murata M. (2023). Knockdown of TFRC suppressed the progression of nasopharyngeal carcinoma by downregulating the PI3K/Akt/mTOR pathway. Cancer Cell Int..

[127] Yu M., Su D., Yang Y., Qin L., Hu C., Liu R., Zhou Y., Yang C., Yang X., Wang G. (2019). D-T7 peptide-modified PEGylated bilirubin nanoparticles loaded with cediranib and paclitaxel for antiangiogenesis and chemotherapy of glioma. ACS Appl. Mater. Interfaces.

[128] Cervera P., Videau C., Viollet C., Petrucci C., Lacombe J., Winsky-Sommerer R., Csaba Z., Helboe L., Daumas-Duport C., Reubi J.C. (2002). Comparison of somatostatin receptor expression in human gliomas and medulloblastomas. J. Neuroendocrinol..

[129] Sun M.L., Wei J.M., Wang X.W., Li L., Wang P., Li M., Yi C.H. (2007). Paclitaxel-octreotide conjugates inhibit growth of human non-small cell lung cancer cells in vitro. Exp. Oncol..

[130] Ma Y., Zhao W., Li Y., Pan Y., Wang S., Zhu Y., Kong L., Guan Z., Wang J., Zhang L. (2019). Structural optimization and additional targets identification of antisense oligonucleotide G3139 encapsulated in a neutral cytidinyl-lipid combined with a cationic lipid in vitro and in vivo. Biomaterials.

[131] Shen Y., Zhang X.Y., Chen X., Fan L.L., Ren M.L., Wu Y.P., Chanda K., Jiang S.W. (2017). Synthetic paclitaxel-octreotide conjugate reverses the resistance of paclitaxel in A2780/Taxol ovarian cancer cell line. Oncol. Rep..

[132] Thompson E.G., Sontheimer H. (2019). Acetylcholine Receptor Activation as a Modulator of Glioblastoma Invasion. Cells.

[133] Egleton R.D., Brown K.C., Dasgupta P. (2008). Nicotinic acetylcholine receptors in cancer: Multiple roles in proliferation and inhibition of apoptosis. Trends Pharmacol. Sci..

[134] McIntosh J.M., Yoshikami D., Mahe E., Nielsen D.B., Rivier J.E., Gray W.R., Olivera B.M. (1994). A nicotinic acetylcholine receptor ligand of unique specificity, alpha-conotoxin ImI. J. Biol. Chem..

[135] Kim M.S., Ma S., Chelariu-Raicu A., Leuschner C., Alila H.W., Lee S., Coleman R.L., Sood A.K. (2020). Enhanced Immunotherapy with LHRH-R Targeted Lytic Peptide in Ovarian Cancer. Mol. Cancer Ther..

[136] Saad M., Garbuzenko O.B., Ber E., Chandna P., Khandare J.J., Pozharov V.P., Minko T. (2008). Receptor targeted polymers, dendrimers, liposomes: Which nanocarrier is the most efficient for tumor-specific treatment and imaging?. J. Control. Release.

[137] Ghanghoria R., Tekade R.K., Mishra A.K., Chuttani K., Jain N.K. (2016). Luteinizing hormone-releasing hormone peptide tethered nanoparticulate system for enhanced antitumoral efficacy of paclitaxel. Nanomedicine.

[138] Baun C., Naghavi-Behzad M., Hildebrandt M.G., Gerke O., Thisgaard H. (2024). Gastrin-releasing peptide receptor as a theranostic target in breast cancer: A systematic scoping review. Semin. Nucl. Med..

[139] Peng S., Zhan Y., Zhang D., Ren L., Chen A., Chen Z.F., Zhang H. (2023). Structures of human gastrin-releasing peptide receptors bound to antagonist and agonist for cancer and itch therapy. Proc. Natl. Acad. Sci. USA.

[140] Arap M.A., Lahdenranta J., Mintz P.J., Hajitou A., Sarkis A.S., Arap W., Pasqualini R. (2004). Cell surface expression of the stress response chaperone GRP78 enables tumor targeting by circulating ligands. Cancer Cell.

[141] Zhang W., Wan L., Han M., Guo W., Wang Z., Zhang X., Liu X., Wang J., Mao Y. (2024). Nose-to-brain drug delivery by HS15 micelles for brain targeting of insoluble drug. Acta Mater. Medica.

[142] Huang Y., Ma Y., Guo Y., Zou L., Jin H., Zhong L., Wu Y., Zhang L., Yang Z. (2014). Exploring directional invasion of serum nuclease into siRNA duplexes by asymmetrical terminal modifications. ChemMedChem.

[143] Ma Y., Liu S., Wang Y., Zhao Y., Huang Y., Zhong L., Guan Z., Zhang L., Yang Z. (2017). Isonucleotide incorporation into middle and terminal siRNA duplexes exhibits high gene silencing efficacy and nuclease resistance. Org. Biomol. Chem..

[144] Li F., Lu J., Liu J., Liang C., Wang M., Wang L., Li D., Yao H., Zhang Q., Wen J. (2017). A water-soluble nucleolin aptamer-paclitaxel conjugate for tumor-specific targeting in ovarian cancer. Nat. Commun..

[145] Lin Q., Ma X., Hu S., Li R., Wei X., Han B., Ma Y., Liu P., Pang Y. (2021). Overexpression of Nucleolin is a Potential Prognostic Marker in Endometrial Carcinoma. Cancer Manag. Res..

[146] Chen M., Zhou P., Kong Y., Li J., Li Y., Zhang Y., Ran J., Zhou J., Chen Y., Xie S. (2023). Inducible Degradation of Oncogenic Nucleolin Using an Aptamer-Based PROTAC. J. Med. Chem..

[147] Ma Y., Xie D., Chen Z., Shen X., Wu X., Ding F., Ding S., Pan Y., Li F., Lu A. (2024). Advancing targeted combination chemotherapy in triple negative breast cancer: Nucleolin aptamer-mediated controlled drug release. J. Transl. Med..

[148] Hefler L.A., Concin N., Mincham D., Thompson J., Swarte N.B., van Eijkeren M.A., Sie-Go D.M., Hammond I., McCartney A.J., Tempfer C.B. (2002). The prognostic value of immunohistochemically detected CD44v3 and CD44v6 expression in patients with surgically staged vulvar carcinoma: A multicenter study. Cancer.

[149] Wachowiak R., Rawnaq T., Metzger R., Quaas A., Fiegel H., Kahler N., Rolle U., Izbicki J.R., Kaifi J., Till H. (2008). Universal expression of cell adhesion molecule NCAM in neuroblastoma in contrast to L1: Implications for different roles in tumor biology of neuroblastoma?. Pediatr. Surg. Int..

[150] Xue F., Lin X., Cai Z., Liu X., Ma Y., Wu M. (2021). Doxifluridine-based pharmacosomes delivering mir-122 as tumor microenvironments-activated nanoplatforms for synergistic treatment of hepatocellular carcinoma. Colloids Surf. B Biointerfaces.

[151] Lv L., Li X., Qian W., Li S., Jiang Y., Xiong Y., Xu J., Lv W., Liu X., Chen Y. (2020). Enhanced anti-glioma efficacy by borneol combined with CGKRK-modified paclitaxel self-assembled redox-sensitive nanoparticles. Front. Pharmacol..

[152] de Graauw M., van Miltenburg M.H., Schmidt M.K., Pont C., Lalai R., Kartopawiro J., Pardali E., Le Devedec S.E., Smit V.T., van der Wal A. (2010). Annexin A1 regulates TGF-beta signaling and promotes metastasis formation of basal-like breast cancer cells. Proc. Natl. Acad. Sci. USA.

[153] McArthur S., Cristante E., Paterno M., Christian H., Roncaroli F., Gillies G.E., Solito E. (2010). Annexin A1: A central player in the anti-inflammatory and neuroprotective role of microglia. J. Immunol..

[154] Lim S.H., Saluja A., Vickers S., Hong J.Y., Kim S.T., Lavania S., Pandey S., Gupta V.K., Velagapudi M.R., Lee J. (2024). The safety and efficacy outcomes of Minnelide given alone or in combination with paclitaxel in advanced gastric cancer: A phase I trial. Cancer Lett..

[155] Liu Y., Liang J., Zhu R., Yang Y., Wang Y., Wei W., Li H., Chen L. (2024). Application of PROTACs in Target Identification and Target Validation. Acta Mater. Medica.

[156] Zhou Y., Xu S., López-Carrobles N., Ding D., Liu X., Menéndez-Arias L., Zhan P. (2023). Recent advances in the molecular design and applications of proteolysis targeting chimera-based multi-specific antiviral modality. Acta Mater. Medica.

[157] Dong W., Lin M., Zhang R., Sun X., Li H., Liu T., Xu Y., Lv L. (2024). d-mannose targets PD-1 to lysosomal degradation and enhances T cell-mediated anti-tumor immunity. Cancer Lett..

[158] Liu J.K., Lubelski D., Schonberg D.L., Wu Q., Hale J.S., Flavahan W.A., Mulkearns-Hubert E.E., Man J., Hjelmeland A.B., Yu J. (2014). Phage display discovery of novel molecular targets in glioblastoma-initiating cells. Cell Death Differ..

[159] Wynendaele E., Verbeke F., Stalmans S., Gevaert B., Janssens Y., Van De Wiele C., Peremans K., Burvenich C., De Spiegeleer B. (2015). Quorum Sensing Peptides Selectively Penetrate the Blood-Brain Barrier. PLoS ONE.

[160] Kandasamy P., Mori S., Matsuda S., Erande N., Datta D., Willoughby J.L.S., Taneja N., O’Shea J., Bisbe A., Manoharan R.M. (2023). Metabolically Stable Anomeric Linkages Containing GalNAc-siRNA Conjugates: An Interplay among ASGPR, Glycosidase, and RISC Pathways. J. Med. Chem..

[161] Ran D., Mao J., Zhan C., Xie C., Ruan H., Ying M., Zhou J., Lu W.L., Lu W. (2017). d-Retroenantiomer of quorum-sensing peptide-modified polymeric micelles for brain tumor-targeted drug delivery. ACS Appl. Mater. Interfaces.

[162] Han M., Wang H., Zhang H.T., Han Z. (2012). The PDZ protein TIP-1 facilitates cell migration and pulmonary metastasis of human invasive breast cancer cells in athymic mice. Biochem. Biophys. Res. Commun..

[163] Wang H., Han M., Whetsell W., Wang J., Rich J., Hallahan D., Han Z. (2014). Tax-interacting protein 1 coordinates the spatiotemporal activation of Rho GTPases and regulates the infiltrative growth of human glioblastoma. Oncogene.

[164] Yao J.-F., Yang H., Zhao Y.-Z., Xue M. (2018). Metabolism of peptide drugs and strategies to improve their metabolic stability. Curr. Drug Metab..

[165] Puente X.S., Gutiérrez-Fernández A., Ordóñez G.R., Hillier L.W., López-Otín C. (2005). Comparative genomic analysis of human and chimpanzee proteases. Genomics.

[166] Tugyi R., Mezö G., Fellinger E., Andreu D., Hudecz F. (2005). The effect of cyclization on the enzymatic degradation of herpes simplex virus glycoprotein D derived epitope peptide. J. Pept. Sci. Off. Publ. Eur. Pept. Soc..

[167] Di L. (2015). Strategic approaches to optimizing peptide ADME properties. AAPS J..

[168] Ballarotto M., Willems S., Stiller T., Nawa F., Marschner J.A., Grisoni F., Merk D. (2023). De Novo Design of Nurr1 Agonists via Fragment-Augmented Generative Deep Learning in Low-Data Regime. J. Med. Chem..

